# Yiqi Jiedu Huayu Decoction Alleviates Renal Injury in Rats With Diabetic Nephropathy by Promoting Autophagy

**DOI:** 10.3389/fphar.2021.624404

**Published:** 2021-04-12

**Authors:** Chen Xuan, Yu-Meng Xi, Yu-Di Zhang, Chun-He Tao, Lan-Yue Zhang, Wen-Fu Cao

**Affiliations:** ^1^ Department of Combination of Chinese and Western Medicine, The First Affiliated Hospital of Chongqing Medical University, Chongqing, China; ^2^ Chongqing Key Laboratory of Traditional Chinese Medicine for Prevention and Cure of Metabolic Diseases, Chongqing, China; ^3^ College of Traditional Chinese Medicine, Chongqing Medical University, Chongqing, China

**Keywords:** yiqi jiedu huayu decoction, diabetic nephropathy, renal fibrosis, podocyte, autophagy

## Abstract

Diabetic nephropathy (DN), a common microvascular complication of diabetes, is one of the main causes of end-stage renal failure (ESRD) and imposes a heavy medical burden on the world. Yiqi Jiedu Huayu decoction (YJHD) is a traditional Chinese medicine formula, which has been widely used in the treatment of DN and has achieved stable and reliable therapeutic effects. However, the mechanism of YJHD in the treatment of DN remains unclear. This study aimed to investigate the mechanism of YJHD in the treatment of DN. Sprague-Dawley rats were randomly divided into a normal control group, a diabetic group, an irbesartan group, and three groups receiving different doses of YJHD. Animal models were constructed using streptozotocin and then treated with YJHD for 12 consecutive weeks. Blood and urine samples were collected during this period, and metabolic and renal function was assessed. Pathological kidney injury was evaluated according to the kidney appearance, hematoxylin-eosin staining, Masson staining, periodic-acid Schiff staining, periodic-acid Schiff methenamine staining, and transmission electron microscopy. The expression levels of proteins and genes were detected by immunohistochemistry, western blotting, and real-time qPCR. Our results indicate that YJHD can effectively improve renal function and alleviate renal pathological injury, including mesangial matrix hyperplasia, basement membrane thickening, and fibrosis. In addition, YJHD exhibited podocyte protection by alleviating podocyte depletion and morphological damage, which may be key in improving renal function and reducing renal fibrosis. Further study revealed that YJHD upregulated the expression of the autophagy-related proteins LC3II and Beclin-1 while downregulating p62 expression, suggesting that YJHD can promote autophagy. In addition, we evaluated the activity of the mTOR pathway, the major signaling pathway regulating the level of autophagy, and the upstream PI3K/Akt and AMPK pathways. YJHD activated the AMPK pathway while inhibiting the PI3K/Akt and mTOR pathways, which may be crucial to its promotion of autophagy. In conclusion, our study shows that YJHD further inhibits the mTOR pathway and promotes autophagy by regulating the activity of the PI3K/Akt and AMPK pathways, thereby improving podocyte injury, protecting renal function, and reducing renal fibrosis. This study provides support for the application of and further research into YJHD.

## Introduction

As a chronic metabolic disease, diabetes mellitus has rapidly become one of the world’s most important health problems. According to the International Diabetes Federation, the number of people with diabetes worldwide will reach 592 million by 2035, compared with 382 million by 2013 (Shi and Hu, 2014). Diabetic nephropathy (DN), a common microvascular complication caused by diabetes, has brought a heavy medical burden to the world. According to statistics, the average medical expenditure of DN patients is 50% higher than that of simple diabetes patients (International Diabetes Federation, 2017). DN is a major cause of end-stage renal failure (ESRD) (Molitch et al., 2004). ESRD is difficult to reverse, so it is particularly important to study the pathogenesis of DN and develop effective early treatment methods.

The pathogenesis of DN is not fully understood. Known pathological lesions in DN include mesangial dilatation, basement membrane thickening, and renal fibrosis. It is worth noting that the development of renal fibrosis is an important reason for the continuous decline of renal function in DN and eventually into ESRD (Kim and Park, 2017; Rao et al., 2019). Podocytes are an important and essential component of the glomerular filtration barrier (Mundel and Reiser, 2010). Current studies have shown that podocyte damage and resulting glomerular filtration barrier defects are important causes of persistent proteinuria and renal fibrosis in DN (Pagtalunan et al., 1997). As terminally differentiated cells, podocytes are difficult to regenerate once damaged. Podocytes have a high basal level of autophagy, which is critical for maintaining their homeostasis in the face of various stimuli (Lenoir et al., 2015; Tagawa et al., 2016). Autophagy is a lysosome-mediated intracellular protein degradation pathway that plays an important role in maintaining intracellular homeostasis and cell integrity (Yorimitsu and Klionsky, 2005). Unfortunately, however, a large number of studies have shown that the autophagic activity of podocytes is inhibited in DN (Kume et al., 2014; Tagawa et al., 2016). Autophagy is mainly regulated by the mTOR pathway, and current studies have shown that selective inhibition of mTOR pathway activity can promote autophagy and improve podocyte damage and renal function (Codogno and Meijer, 2005; Yang et al., 2020). The activity of the mTOR pathway is mainly regulated by the PI3K/Akt and AMPK pathways (Yang and Klionsky, 2010). Therefore, targeting the mTOR pathway may be an appropriate treatment for DN, thereby promoting autophagic activity, improving podocyte injury and renal pathological changes, and restoring renal function.

Currently, the main therapeutic measures for DN include the control of blood glucose, lipids, blood pressure, and the application of vasoactive drugs. Among them, as an angiotensin II receptor blocker (ARB), irbesartan is a representative drug in the clinical treatment of diabetic nephropathy, which can effectively reduce the proteinuria of patients in clinical trials. (Masuda et al., 2009; Nakamura et al., 2014). These methods delay the development of DN to some extent but do not target renal injury, making it difficult to completely prevent renal failure (Sulaiman, 2019). Therefore, it is necessary to develop safe and effective treatments. Traditional Chinese medicine (TCM) has been used to treat diabetes mellitus and diabetic complications for centuries and has attracted more attention in recent years due to its remarkable clinical efficacy. Yiqi Jiedu Huayu decoction (YJHD), a modified traditional Chinese prescription, has been used in clinical treatment of DN. YJHD consists of seven herbs. Many of these herbs, including *Astragalus mongholicus Bunge*, *Pueraria montana var. lobata (willd.) Maesen and S. M. Almeida ex Sanjappa and Predeep*, *Coptis chinensis Franch*, and *Morus alba L*, have been proven to have therapeutic effects on diabetes mellitus and diabetic complications (Yang et al., 2016; Chen et al., 2018; Thaipitakwong et al., 2018; Zhang et al., 2019; Xiao et al., 2020). Owing to the complexity of components and targets of traditional Chinese medicine prescriptions, the mechanism of YJHD in treating DN remains unclear. YJHD is considered a good candidate for treating DN, and therefore its underlying mechanism of action warrants further investigation.

In this study, we first identified the main components of YJHD by high-performance liquid chromatography (HPLC). We then evaluated the effects of YJHD on diabetic symptoms and renal function in DN rats. We hypothesized that YJHD could regulate the activities of the PI3K/Akt, AMPK, and mTOR pathways, promote autophagy, improve podocyte injury and renal pathological changes, and improve renal function.

## Materials and Methods

### Drugs and Reagents

This study used traditional Chinese medicine granules of single herbs provided by Tianjiang Pharmaceutical (Jiangyin, China) to ensure accurate dosage. Each herb has gone through a series of processes including decoction, extraction, concentration, and drying, and finally prepared into granules (Yu et al., 2020). These granules were identified by Dr Wenfu Cao of Chongqing Medical University and preserved in Chongqing Key Laboratory of Traditional Chinese Medicine for Prevention and Cure of Metabolic Diseases. Irbesartan was purchased from Sanofi-Aventis (Paris, France). Streptozotocin (STZ) was purchased from Sigma-Aldrich (St. Louis, MO, United States). Sodium citrate buffer was purchased from Solarbio Bioscience and Technology (Beijing, China). The serum creatinine (Cr) detection kit, serum urea nitrogen (BUN) detection kit, urine protein detection kit, serum alanine aminotransferase (ALT) detection kit and serum aspartate aminotransferase (AST) detection kit were provided by Nanjing Jiancheng Bioengineering Institute (Nanjing, China). TRIzol, PrimeScript RT reagent Kit with gDNA Eraser (RR047A), and TB Green Premix Ex Taq II (RR820A) were from Takara Bio (Kusatsu, Japan). All primers for qPCR were synthesized by Tsingke Biology Technology (Beijing, China). Acetonitrile, methanol, and formic acid (LC-MS grade) were purchased from CNW Technologies (Dusseldorf, Germany).

Antibodies against p62 (5114), Beclin-1 (3495), Akt (3063), p-Akt (Ser473, 4060), LKB1 (3047), AMPK (2532), p-AMPK (Thr172, 2535), p-mTOR (Ser2448, 5536), and β-actin (4970) were from Cell Signaling Technology (Danvers, MA, United States). Antibodies against fibronectin (ab2413), collagen IV (ab6586), LC3I/II (ab128025), mTOR (ab2732), and PI3K (ab191606) were provided by Abcam (Cambridge, United Kingdom). Antibodies to ULK1 (A00581-1), nephrin (BA1669), and podocin (BA1688) were provided by Boster Bio (Pleasanton, CA, United States). The antibody to IRS1 (BS-0172R) was purchased from Bioss (Beijing, China). The antibody to p-ULK1 (Ser757, 12871) was provided by Signalway Antibody (College Park, MD, United States).

### Preparation of Yiqi Jiedu Huayu Decoction

YJHD consists of seven different herbs, including Huang Qi (*Astragalus mongholicus Bunge*), GeGen (*Pueraria montana var. lobata (Willd.) Maesen and S. M. Almeida ex Sanjappa and Predeep*), HuangQin (*Scutellaria baicalensis Georgi*), HuangLian (*Coptis chinensis Franch*), SanLeng (*Sparganium stoloniferum (Buch.-Ham. ex Graebn.) Buch.-Ham. ex Juz*), JiangHuang (*Curcuma longa L*), and SangYe (*Morus alba L*). The herbal information and composition ratio are shown in Table 1. The daily dose of YJHD granules in adults is 11.5 g. According to the conversion ratio of surface area between rat and humans (6.3), the daily dose for rats was calculated to be 1.2 g/kg. This dose was used as the medium dose. The low and high doses of YJHD were 0.6 and 2.4 g/kg, respectively (Wang et al., 2020). According to this ratio, granules of single herbs were mixed and dissolved in double-distilled water to make YJHD oral liquids at different concentrations (Chen et al., 2021). Part of the YJHD oral liquids was stored at −80°C for UPLC-QTOF-MS analysis.

**TABLE 1:** Composition of Yiqi Jiedu Huayu decoction.

Scientific name	Pinyin name	Plant part used	Batch number	Adult daily dose of granules (g)	Corresponding herb dose (g)	Composition (%)
*Astragalus mongholicus Bunge*	HuangQi	Root	19040231	4.5	30	31.3
*Pueraria montana var. lobata (Willd.) Maesen and S.M.Almeida ex Sanjappa & Predeep*	GeGen	Tuber	19070871	2	20	20.8
*Scutellaria baicalensis Georgi*	HuangQin	Root	19041911	2	10	10.4
*Coptis chinensis Franch*	HuangLian	Rhizome	19041621	1	6	6.3
*Sparganium stoloniferum (Buch.-Ham. ex Graebn.) Buch.-Ham. ex Juz*	SanLeng	Tuber	18082331	0.5	10	10.4
*Curcuma longa L*	JiangHuang	Tuber	19030331	0.5	10	10.4
*Morus alba L*	SangYe	Leaf	19011351	1	10	10.4

### UPLC-QTOF-MS Analysis

The samples of YJHD were thawed in ice water, vortexed for 30 s and centrifuged at 12,000 rpm at 4°C for 10 min. A 300-μl aliquot of each individual sample was precisely transferred to an Eppendorf tube. After the addition of 1,000 μl extracting solution (methanol:water = 4:1, v/v, including internal standard (IS) at a ratio of 1,000:10), all samples were vortexed for 30 s, sonicated for 10 min in an ice-water bath, incubated at −80°C for 1 h, and centrifuged at 12,000 rpm at 4°C for 10 min. A 400-μl aliquot of the supernatant was passed through a 0.22-μm filter membrane and then transferred to an auto-sampler vial for ultra-high-performance liquid chromatography tandem mass spectrometry (UHPLC-MS/MS) analysis.

The LC separation was performed on a UPLC BEH C18 column (1.7 μm × 2.1 mm × 100 mm, Waters, United States); 0.1% formic acid (phase A) and acetonitrile (phase B) were used as the mobile phases. The gradient elution was programmed as follows: 0–3.5 min (5–15% B), 3.5–6 min (15–30% B), 6–12 min (30–70% B), 12–18 min (70–100% B), and 18–25 min (100% B). The flow rate was 400 μl/min. The injected sample volume was 5 μl. This Xcalibur system was connected to Q Exactive Focus mass spectrometer (Thermo Fisher Scientific, United States). Sheath gas flow rate: 45 Arb, Aux gas flow rate: 15 Arb, Capillary temperature: 400°C, Full ms resolution: 70,000, MS/MS resolution: 17,500, Collision energy: 15/30/45 in NCE mode, Spray Voltage: 4.0 kV (positive) and −3.6 kV (negative). Mass spectra were imported raw using XCMS software. Retention time correction, peak identification, peak extraction, peak integration, and peak alignment were performed. Material identification of peaks containing MSMS data was performed using the secondary mass spectrometry database provided by Shanghai BIOTREE biotech Co., Ltd. and the corresponding cleavage law matching method.

### Animal Experiments

Ten-week-old male Sprague-Dawley rats (250 ± 30 g) were used as research subjects. All animals were provided by the Laboratory Animal Center of Chongqing Medical University and kept in a specific-pathogen-free level of the center (SYXK 2018-0003). All animal experiments were conducted in compliance with the National Institute of Health Guide for the Care and Use of Laboratory Animals and were approved by the Ethics Committee of The First Affiliated Hospital of Chongqing Medical University.

After 2 weeks of adaptive feeding, all rats were randomly divided into seven groups (*n* = 10): 1) normal control group; 2) normal control + YJHD-H group, oral high-dose YJHD; 3) diabetic group; 4) irbesartan group; 5) YJHD-L group, oral low-dose YJHD; 6) YJHD-M group, oral medium-dose YJHD; and 7) YJHD-H group, oral high-dose YJHD. STZ injection was prepared by dissolving STZ in 0.1 M sodium citrate buffer (pH 4.5). All rats except the normal control group and normal control + YJHD-H group received a single intraperitoneal injection of 60 mg/kg STZ injection to establish a diabetic model. Meanwhile, rats in the normal control group and normal control + YJHD-H group were intraperitoneally injected with the corresponding dose of sodium citrate buffer to ensure the reliability of the experiment. One week after STZ injection, the animal model was evaluated by detecting fasting blood glucose twice per rat. When both fasting blood glucose tests were greater than 16.7 mmol/l, the diabetic model was considered to be established; otherwise, the rat was excluded from the experiment (Han et al., 2017a; Xu et al., 2017). Drug intervention was initiated 1 week after confirmation of the model. Rats in the normal control + YJHD-H group, irbesartan group, YJHD-L group, YJHD-M group, and YJHD-H group received corresponding oral liquid by gavage. Rats in the normal control and Diabetic groups received the corresponding dose of double-distilled water by gavage. The drug was given once a day for 12 weeks (Xu et al., 2017). At the end of the drug intervention, rats were anesthetized with isoflurane and blood was taken through the orbital vein. Rats were then sacrificed by isoflurane anesthetics and high concentrations of carbon dioxide. Kidneys were immediately isolated, their appearance was recorded, and they were weighed.

### Detection of Metabolic and Biochemical Indicators

Beginning at the start of the drug intervention, the body weight, 24-h food intake, and 24-h water intake of rats were recorded every 4 weeks. In addition, 24-h urine was collected through metabolic cages every 4 weeks, and urine volume was recorded. Urinary protein concentration was measured according to the manufacturer’s instructions. Then, 24-h urinary protein quantification was calculated by urine volume and urinary protein concentration. Blood was collected through the orbital vein at the end of the drug intervention, centrifuged at 3,000 rpm for 10 min, and then the upper serum was collected for later use. Blood glucose, Cr, BUN, ALT, and AST were assessed according to the manufacturer’s instructions.

### Histological and Immunohistochemical Assays

#### Kidney Tissue Pretreatment

Kidney tissues were cut into small pieces, with one part fixed in 4% paraformaldehyde for 24 h, dehydrated, embedded in paraffin, and cut into 6-μm sections for hematoxylin-eosin (HE), Masson, periodic-acid Schiff (PAS), and PAS methenamine (PASM) staining and immunohistochemical (IHC) experiments according to the manufacturer’s instructions. The other part was further cut into small pieces of 1 mm³ and fixed in glutaraldehyde for analysis by transmission electron microscopy.

#### Hematoxylin-Eosin Staining

The sections were dip-stained with hematoxylin solution for 5 min, rinsed with distilled water, differentiated with hematoxylin differentiation solution, rinsed with distilled water, treated with Scott’s Tap Water Bluing solution, and again rinsed with distilled water. The sections were then immersed in 85% ethanol for 5 min, 95% ethanol for 5 min, and stained with eosin dye for 5 min. Sections were dehydrated with ethanol and xylene in turn and then sealed with neutral gum. All the sections were analyzed by microscopy (BX53, Olympus Corporation, Japan).

#### Masson Staining

The sections were soaked in Masson A overnight and then rinsed with distilled water. Masson solution was prepared by mixing Masson B and Masson C in a 1:1 ratio. The sections were then stained with Masson solution for 1 min, rinsed with distilled water, differentiated with 1% hydrochloric acid alcohol, rinsed with distilled water, soaked in Masson D for 6 min, rinsed with distilled water, and finally soaked in Masson E for 1 min and Masson F for 30 s. The sections were rinsed with 1% glacial acetic acid, dehydrated with anhydrous ethanol, and sealed with neutral gum. Following this, the sections were analyzed by microscopy.

#### Periodic Acid-Schiff Staining

The sections were stained with PAS dye solution B for 15 min, rinsed twice with distilled water, stained with PAS A for 30 min in the dark, rinsed for 5 min, stained with PAS C for 30 s, and rinsed with distilled water. The sections were then treated with hydrochloric acid solution and ammonia. Sections were dehydrated with ethanol and xylene in turn and then sealed with neutral gum. All the sections were analyzed by microscopy.

#### Periodic Acid-Silver Methenamine Staining

The sections were soaked in PASM staining solution A overnight and rinsed with distilled water. PASM staining solutions D and C were mixed into a PASM stock solution at a 20:1 ratio. PASM stock solution, distilled water, and PASM staining solution E were proportionally mixed to form a working solution. Sections were then acidified in PAS staining solution B. The working solution was added to the sections drop by drop, and the sections were incubated with a cover for 40 min at 57°C. They were then treated with PASM staining solution F, dehydrated with ethanol and xylene in turn, and then sealed with neutral gum. Thereafter, the sections were analyzed by microscopy.

#### Immunohistochemical Experiments

The sections were incubated with citrate antigen retrieval solution for 20 min at 95°C. Thereafter, the sections were incubated with anti-fibronectin (1:100), anti-collagen IV (1:100), anti-podocin (1:100), and anti-nephrin (1:100) antibodies overnight and then with a secondary antibody for 50 min. The cumulative optical density was collected and calculated with Image-Pro Plus software (Media Cybernetics, United States).

#### Kidney Ultrastructural Morphology

Fixed kidney segments were rinsed four times with a 1 M phosphate-buffered solution, 15 min each time. Segments then were dehydrated by graded ethanol, immersed in isoamyl acetate, and routinely dried and treated. Kidney ultrastructural images were observed under transmission electron microscopy (JEM-1400PLUS, JEOL Ltd, Japan).

### Real-Time Quantitative PCR Analysis

mRNA expression levels of collagen IV, fibronectin, p62, Beclin-1, mTOR, and ULK1 in kidneys were detected by real-time quantitative PCR. According to the manufacturers’ protocols, total RNA was extracted from kidney tissue using TRIzol and cDNA was synthesized by reverse transcription, cDNA and TB Green Premix Ex Taq II were premixed for PCR. The thermal reaction cycle was as follows: 95°C for 30 s, and then 40 cycles of 95°C for 5 s and 60°C for 30 s (CFX Connect, Bio-Rad, United States). β-actin was used as a housekeeping gene to standardize Ct values. Fold changes of mRNA expression were calculated by relative quantification (2^−ΔΔCt^). Primer sequences used for PCR are shown in Table 2.

**TABLE 2:** Primer sequences used for PCR.

Gene		Oligonucleotide sequence
Collagen Ⅳ	Forward	5′-ACA​CAG​TCA​AAC​CAC​AGC​CAA​TCC-3′
	Reverse	5′-TGG​GCG​TGC​TCA​TTT​CCT​TGT​AC-3′
Fibronectin	Forward	5′-CTT​GGT​GCG​CTA​CTC​ACC​TG-3′
	Reverse	5′-ATG​CTG​TTC​GTA​CAC​GCT​GG-3′
p62	Forward	5′-AAG​TTC​CAG​CAC​AGG​CAC​AGA​AG-3′
	Reverse	5′-TCC​CAC​CGA​CTC​CAA​GGC​TAT​C-3′
Beclin-1	Forward	5′-GGC​CAG​ACA​GTG​TTG​TTG​CT-3′
	Reverse	5′-CCC​CAG​AAC​AGT​ACA​ACG​GC-3′
mTOR	Forward	5′-GTG​TGG​CAA​GAG​CGG​CAG​AC-3′
	Reverse	5′-TGT​TGG​CAG​AGG​ATG​GTC​AAG​TTG-3′

### Western Blot Analysis

Proteins from kidney tissues were extracted using radioimmunoprecipitation buffer containing protease inhibitors and phosphatase inhibitors, and protein concentrations were assessed using a bicinchoninic acid protein assay kit. Protein expression levels were detected using antibodies to collagen IV (1:1,000), fibronectin (1:1,000), podocin (1:1,000), nephrin (1:500), Beclin-1 (1:1,000), p62 (1:1,000), LC3 I/II (1:1,000), mTOR (1:2,000), p-mTOR (1:1,000), ULK1 (1:1,000), p-ULK1 (1:1,000), IRS1 (1:1,000), PI3K (1:1,000), Akt (1:1,000), p-Akt (1:1,000), LKB1 (1:1,000), AMPK (1:1,000), and p-AMPK (1:1,000) according to the western blotting experimental protocol. β-actin (1:1,000) was used as a control to keep protein loading consistent. The western blotting process was as follows. First, target proteins were separated by sodium dodecyl sulfate polyacrylamide gel electrophoresis (SDS-PAGE) and transferred to polyvinylidene fluoride (PVDF) membranes. PVDF membranes with target proteins were then blocked with 5% skim milk for 2 h and incubated with primary and secondary antibodies. Finally, enhanced chemiluminescence reagents were added to the PVDF membrane and target proteins were visualized by a chemiluminescence imaging system (Odyssey Fc, LI-COR Biosciences, United States).

### Statistical Analysis

All experiments in this study were repeated three times independently to ensure reliable results. All statistical analyses were performed using SPSS 24.0 software (IBM, United States). The statistical analyses were performed by one-way analysis of variance (ANOVA) followed by a least significant difference (LSD) test or Dunnett’s T3 test. All data were expressed as means ± standard deviations. When *p* < 0.05, the result was considered to be statistically significant. GraphPad Prism 8.0.1 (GraphPad Software, United States) was used for image production.

## Results

### Components Analysis of Yiqi Jiedu Huayu Decoction

**FIGURE 1:**
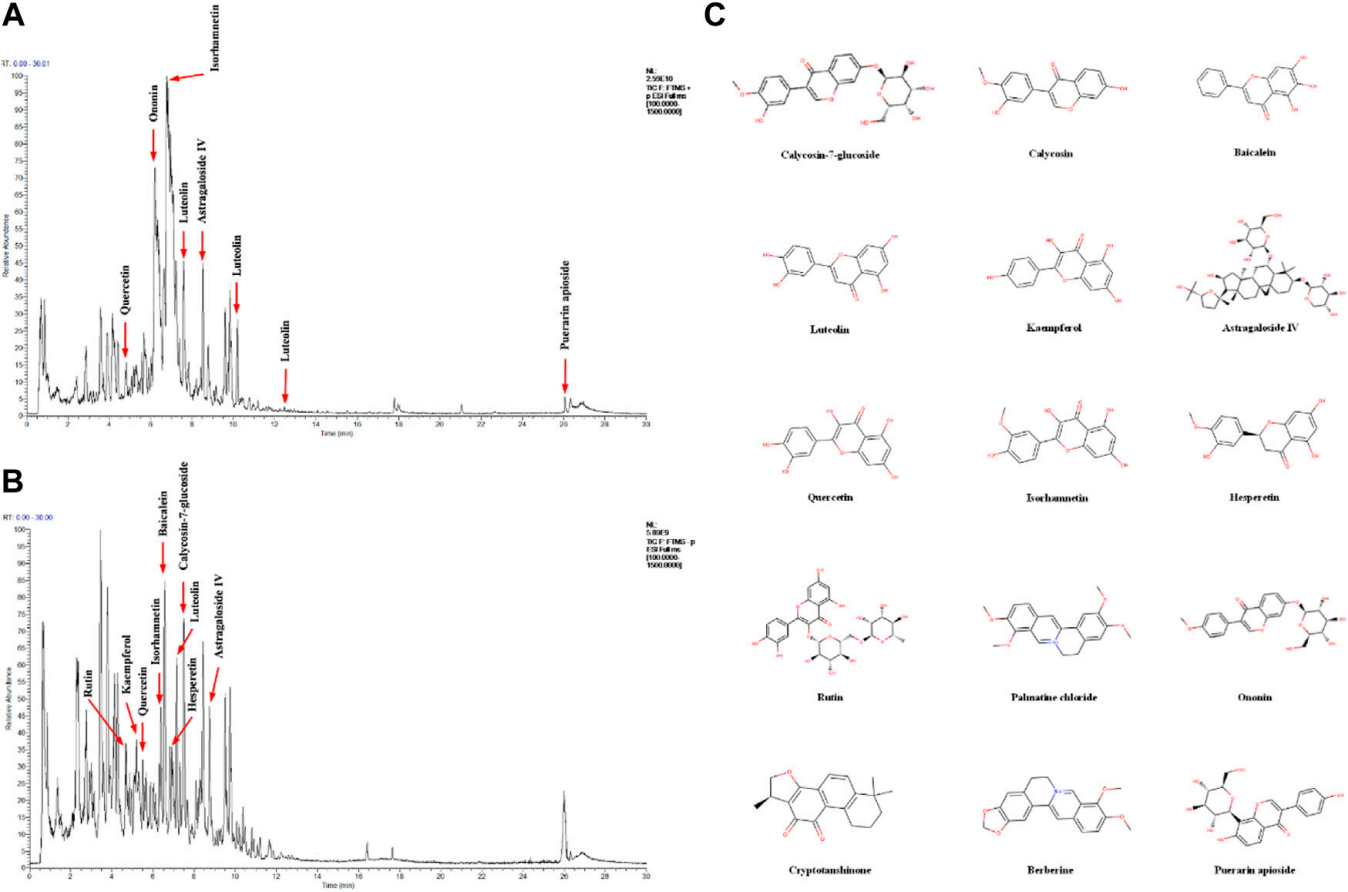
Identification of chemical components of Yiqi Jiedu Huayu decoction (YJHD). YJHD samples were examined by UHPLC–MS/MS. Total ion chromatography in positive **(A)** and negative **(B)** ion modes for YJHD samples are shown. **(C)** Molecular structure of constituents. UHPLC–MS/MS, ultra-high performance liquid chromatography tandem mass spectrometry.

To identify the major chemical components, YJHD samples were analyzed by UHPLC-MS/MS. The total positive (Figure 1A) and negative (Figure 1B) ion chromatograms of YJHD demonstrated the chemical composition of all compounds. Several components were found in YJHD. As shown in Figure 1C, fifteen compounds were distinguished: 1) calycosin-7-glucoside, 2) kaempferol, 3) baicalein, 4) luteolin, 5) calycosin, 6) astragaloside IV, 7) quercetin, 8) isorhamnetin, 9) hesperetin, 10) rutin, 11) palmatine chloride, 12) ononin, 13) cryptotanshinone, 14) berberine, and 15) puerarin apioside.

### Yiqi Jiedu Huayu Decoction Improves Diabetic Symptoms and Protects Renal Function

**FIGURE 2:**
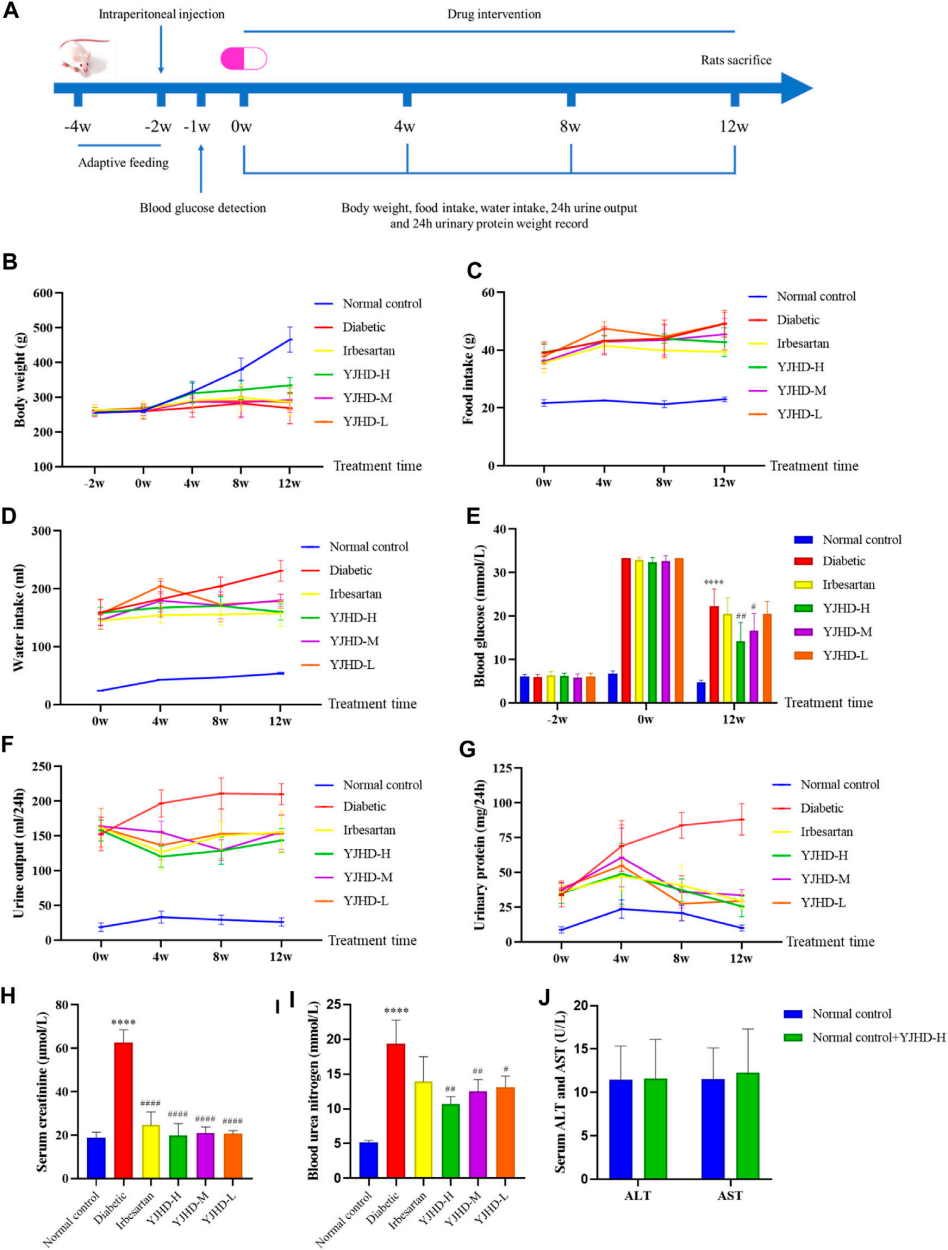
Yiqi Jiedu Huayu decoction (YJHD) improves diabetic symptoms and renal function. **(A)** Timeline of animal experiments. **(B–D)** Changes in body weight and 24-h food and water intake of rats during drug intervention (recorded every 4°weeks). **(E)** Changes in blood glucose before and after drug intervention. **(F, G)** Changes in 24-h urine volume and 24-h urinary protein quantification during drug intervention (measured every 4°weeks). **(H, I)** Serum creatinine (Cr) and blood urea nitrogen (BUN) levels after drug intervention. **(J)** Serum alanine aminotransferase (ALT) and aspartate aminotransferase (AST) levels after drug intervention. The data are expressed as ****p < 0.0001: compared with the normal control group; #*p* < 0.05, ##*p* < 0.01, and ####*p* < 0.0001: compared with the diabetic group, respectively.

Serum ALT and AST levels were detected in rats in the normal control + YJHD-H and normal control groups to evaluate the safety of YJHD. There was no significant difference in serum ALT and AST levels between the normal control + YJHD-H group and the normal control group (*p* > 0.1), indicating that YJHD had no hepatotoxicity (Figure 2J). To verify the effect of YJHD on DN, we recorded the body weight and 24-h food and water intake of rats every 4 weeks, collected 24-h urine, and calculated 24-h urinary protein quantification. At the end of the last drug intervention, we obtained blood samples from rats via the orbital vein to detect blood glucose, Cr, and BUN. As the age increased, the weight of the rats in the normal control group increased, while the rats in the diabetic group appeared to be in growth arrest (*p* < 0.0001). Compared with the diabetic group, the weight of rats in the YJHD-H group significant increased (*p* < 0.01) (Figure 2B). Compared with the normal control group, the 24-h food and water intake of the diabetic, irbesartan, YJHD-L, YJHD-M, and YJHD-H groups were significantly increased (*p* < 0.0001). Compared with the diabetic group, the amount of food and water intake in the irbesartan and YJHD-H groups decreased significantly (*p* < 0.0001), while the changes in the YJHD-L and YJHD-M groups were not significant (Figures 2C,D). The blood glucose level of the normal control group was kept within the normal range. Compared with the normal control group, the blood glucose of the remaining rats was significantly increased (*p* < 0.0001). Compared with the diabetic group, the blood glucose in the YJHD-H (*p* < 0.01) and YJHD-M (*p* < 0.05) groups was significantly lower (Figure 2E).

We evaluated renal function by 24-h urine volume, 24-h urinary protein quantification, serum Cr, and BUN. Compared with the normal control group, the 24-h urine volume, 24-h urine protein quantification, serum Cr, and BUN of the diabetic group rats were significantly increased (*p* < 0.0001). Compared with the diabetic group, YJHD and irbesartan significantly reduced these indicators (*p* < 0.01). The improvement seen in the YJHD-H group was the most obvious (Figures 2F–I). According to these results, YJHD improved diabetic symptoms and renal function, and the effect of high-dose YJHD was the most obvious, so the YJHD-H group was used as a representative group in the following studies.

### Yiqi Jiedu Huayu Decoction Alleviates Renal Pathological Injury

**FIGURE 3:**
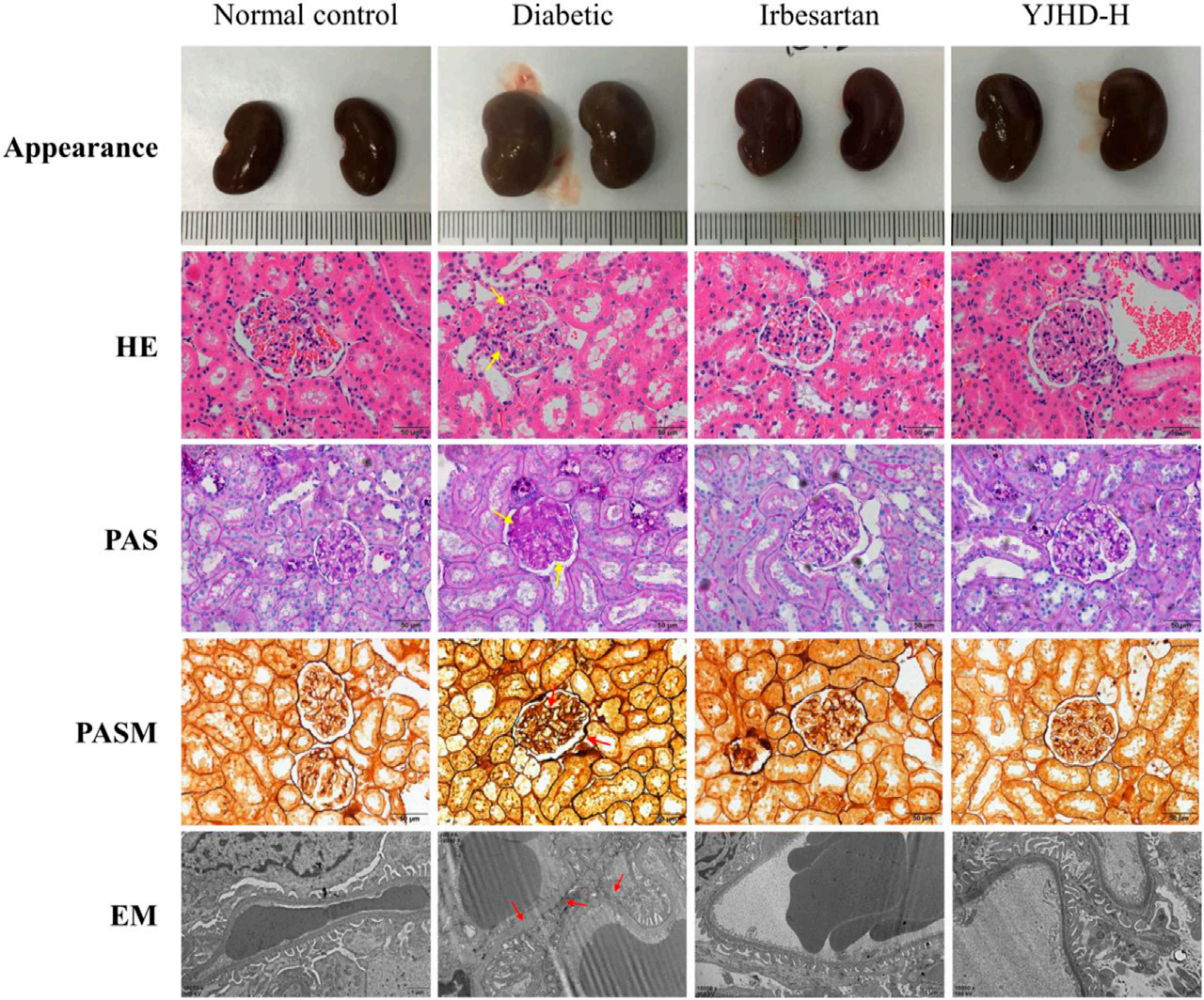
Yiqi Jiedu Huayu decoction (YJHD) attenuated renal histological damage. Renal pathological changes were analyzed by renal appearance; hematoxylin-eosin, periodic-acid Schiff, and periodic-acid Schiff methenamine staining; and transmission electron microscopy. In the diabetic group, the positive parts of the lesions are marked with arrows.

Mesenchymal matrix hyperplasia, basement membrane thickening, and renal fibrosis are considered typical pathological features of DN, both in clinical diagnosis and in animal experiments. In this study, we evaluated the pathological changes of the kidney from different perspectives by renal appearance; HE, PAS, PASM staining; and transmission electron microscopy (Figure 3). The normal control group showed normal renal appearance, no obvious pathological changes in glomeruli and renal tubules, normal podocyte structure, and regular foot processes. Compared with the normal control group, the diabetic group showed enlarged kidneys, generally abnormal appearance of kidneys, expansion of the mesangial area, accumulation of extracellular matrix (ECM), thickening of the basement membrane, abnormal podocyte morphology, and absent foot process fusion. In the diabetic group, the positive parts of the lesions are marked with arrows. Compared with the diabetic group, irbesartan and high-dose YJHD alleviate these pathological injuries.

### Yiqi Jiedu Huayu Decoction Improves Renal Fibrosis

**FIGURE 4:**
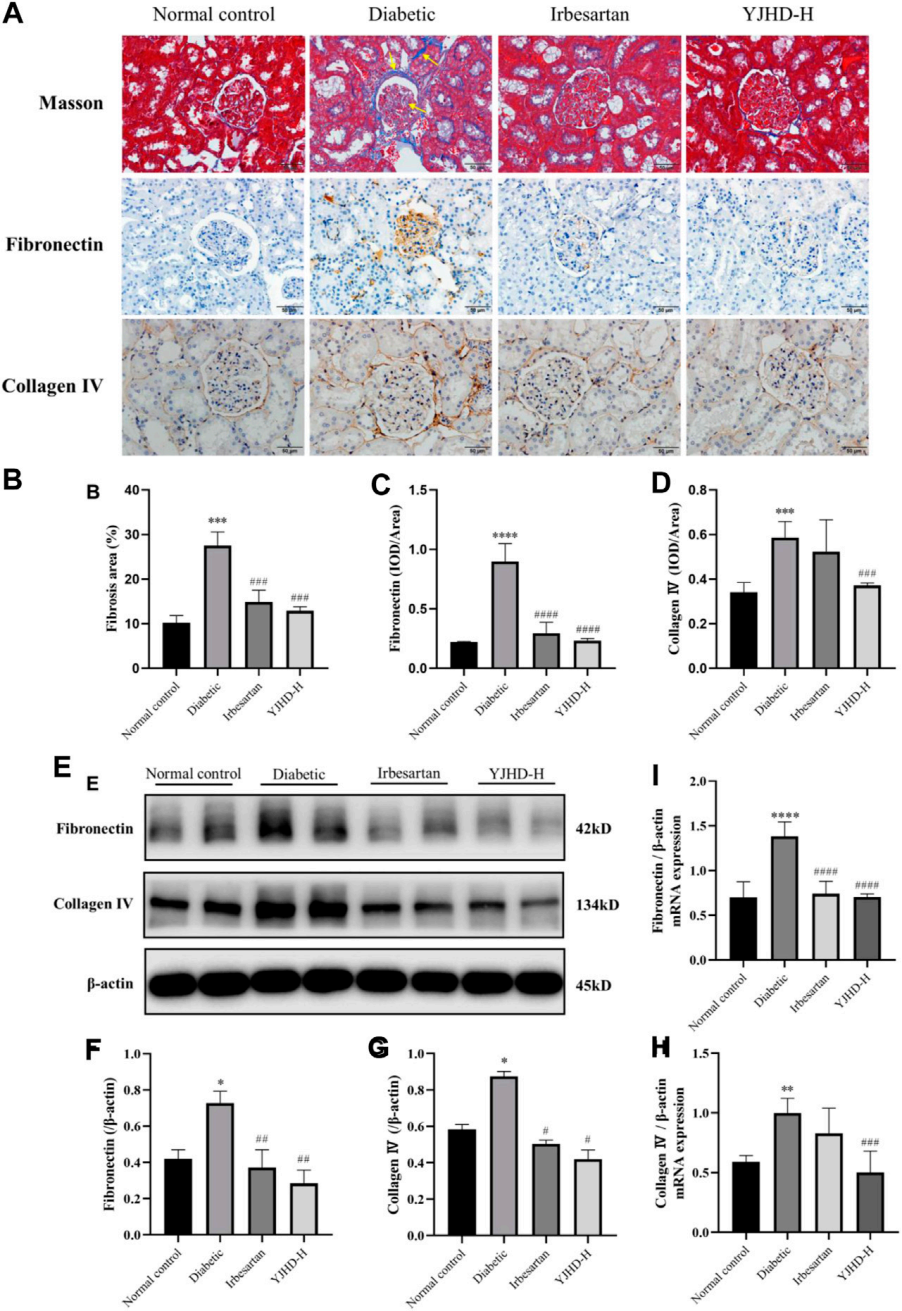
Yiqi Jiedu Huayu decoction (YJHD) improves renal fibrosis. **(A)** Masson staining was used to analyze collagen fibers in the kidney, and immunohistochemistry was used to analyze the expression of fibronectin and collagen IV. In the diabetic group, collagen fibers deposition (arrows) was observed in Masson staining. **(B)** ImageJ software was used to calculate the degree of renal fibrosis. **(C, D)** Image-Pro Plus software was used to statistically analyze immunohistochemical staining results of fibronectin and collagen IV, respectively. **(E)** Protein levels of fibronectin and collagen IV in the kidney were detected by western blotting. **(F, G)** Protein concentration analysis. **(H, I)** mRNA expression levels of fibronectin and collagen IV in kidneys were detected by PCR. Data are expressed as *p < 0.05, **p < 0.01, ***p < 0.001, ****p < 0.0001: compared with the normal control group; #*p* < 0.05, ##*p* < 0.01, ###*p* < 0.001, and ####*p* < 0.0001: compared with the diabetic group, respectively.

Renal fibrosis is an important cause of the decreasing renal function in DN. To evaluate renal fibrosis, we performed Masson staining of kidney tissue and examined levels of collagen IV and fibronectin, the main components of the ECM, by qPCR, western blot, and immunohistochemistry. In Masson staining, red color indicates muscle fibers and blue indicates collagen fibers. Compared with the normal control group, there was a significant accumulation of collagen fibers (arrows) in the kidney tissue of the diabetic group (*p* < 0.001). Compared with the diabetic group, high-dose YJHD significantly reduced the number of collagen fibers and improved renal fibrosis (*p* < 0.001) (Figures 4A,B). In addition, collagen IV and fibronectin expression in the Diabetic group increased significantly at both mRNA and protein levels compared with the normal control group (*p* < 0.05). High-dose YJHD significantly reduced collagen IV and fibronectin expression in the kidney compared with the diabetic group (*p* < 0.05) (Figures 4A–H). These results suggest that high-dose YJHD can effectively ameliorate renal fibrosis in DN rats.

### YJHD Alleviated Podocyte Injury

**FIGURE 5:**
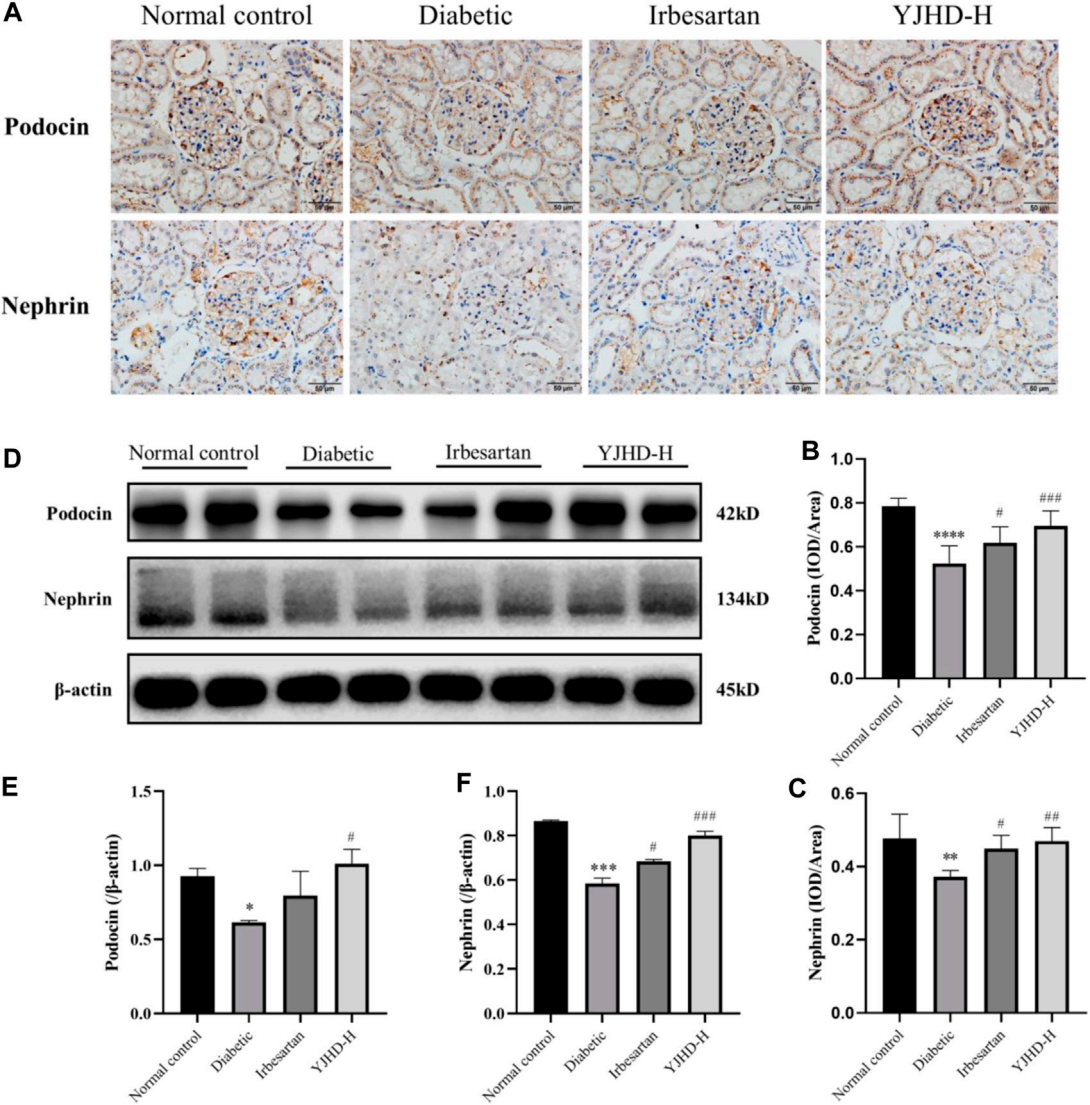
Yiqi Jiedu Huayu decoction (YJHD) alleviates podocyte injury. **(A)** Podocin and nephrin expression were detected by immunohistochemistry. **(B, C)** Immunohistochemical staining results of podocin and nephrin were analyzed by Image-Pro Plus software. **(D)** Protein levels of podocin and nephrin in kidneys were detected by western blot. **(E, F)** Protein concentration analysis. Data are expressed as *p < 0.05, **p < 0.01, ***p < 0.001, ****p < 0.0001: compared with the normal control group; #*p* < 0.05, ##*p* < 0.01, ###*p* < 0.001: compared with the diabetic group.

Podocytes are important structures in maintaining renal filtration function. Podocyte injury is closely related to proteinuria and renal fibrosis in DN. Transmission electron microscopy showed that, compared with the normal control group, podocytes in the diabetic group suffered from morphological damage and foot process fusion. Compared with the diabetic group, podocytes in the YJHD-H group showed relatively normal cell structure (Figure 3). To evaluate the effect of YJHD on podocytes more accurately, we analyzed the expression of podocin and nephrin by western blot and immunohistochemistry. Compared with the normal control group, podocin and nephrin in the diabetic group decreased significantly (*p* < 0.05), indicating that the number of normal podocytes in the diabetic group decreased. Compared with the diabetic group, irbesartan and high-dose YJHD increased podocin and nephrin expression and alleviated podocyte injury (*p* < 0.05) (Figures 5A–F).

### Yiqi Jiedu Huayu Decoction Promotes Autophagy

**FIGURE 6:**
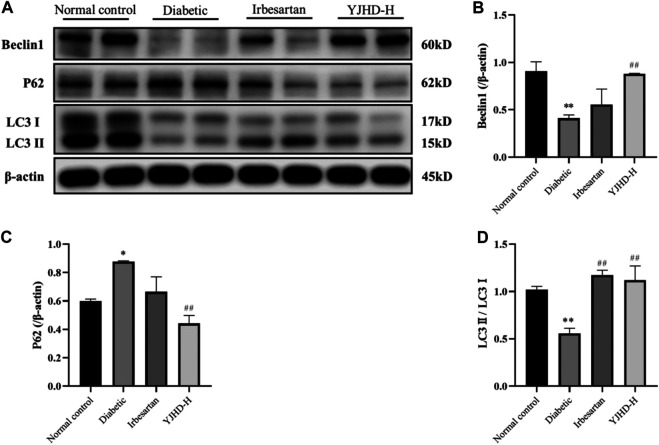
Effects of Yiqi Jiedu Huayu decoction (YJHD) on autophagy. **(A)** Protein levels of Beclin-1, p62, and LC3II were detected by western blotting. **(B–D)** Protein concentration analysis. Data are expressed as *p < 0.05, **p < 0.01: compared with the normal control group, respectively; #*p* < 0.05, ##*p* < 0.01: compared with the diabetic group.

To evaluate the effects of YJHD on autophagy, we examined the expression of Beclin-1, p62, and LC3II mRNA and protein in the kidney. These proteins play an important role in autophagy and can be used to assess autophagy fluxes. Compared with the normal control group, Beclin-1 and LC3II expression decreased and p62 expression increased in the diabetic group (*p* < 0.05), indicating that autophagy in the diabetic group was inhibited. Compared with the diabetic group, high-dose YJHD significantly upregulated Beclin-1 and LC3II expression and downregulated p62 expression (*p* < 0.05) (Figures 6A–D). This suggests that high-dose YJHD promotes autophagy, which may be responsible for its effects in the alleviation of podocyte injury.

### Effects of Yiqi Jiedu Huayu Decoction on mTOR Pathway

**FIGURE 7:**
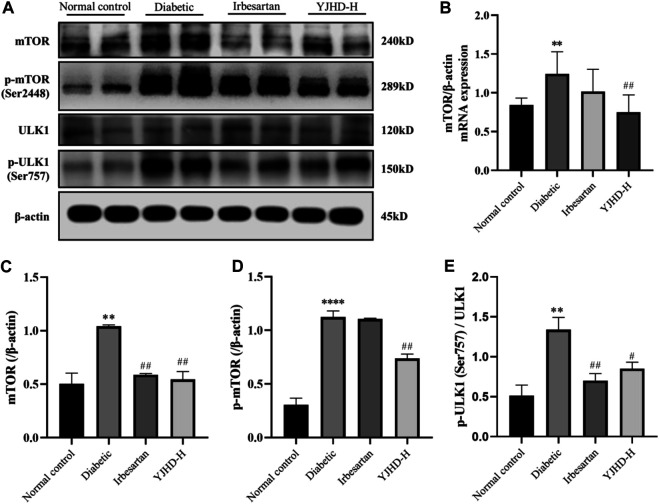
Effects of Yiqi Jiedu Huayu decoction (YJHD) on mTOR pathway. **(A)** Protein levels of mTOR, p-mTOR, ULK1, and p-ULK1 were detected by western blotting. **(B)** mRNA expression of mTOR was detected by PCR. **(C–E)** Protein concentration analysis. Data are expressed as *p < 0.05, **p < 0.01, and ***p < 0.001: compared with the normal control group; ##*p* < 0.01: compared with the diabetic group.

The mTOR pathway is a key pathway regulating autophagy. Activation of the mTOR pathway is associated with inhibition of autophagy. We examined the expression of mTOR, p-mTOR, and downstream ULK1 and p-ULK1 in the kidney. Compared with the normal control group, mTOR and p-mTOR in kidneys of the diabetic group was significantly increased (*p* < 0.05). Compared with the diabetic group, high-dose YJHD significantly downregulated mTOR and p-mTOR expression (*p* < 0.01) (Figures 7A–D). Activated mTOR inhibits autophagy by phosphorylating ULK1. Compared with the normal control group, p-ULK1 expression in the kidneys of the diabetic group was significantly increased (*p* < 0.01). Compared with the diabetic group, high-dose YJHD significantly reduced the expression of p-ULK1 (*p* < 0.05) (Figures 7A,E). This suggests that high-dose YJHD can inhibit activation of the mTOR pathway.

### Effects of Yiqi Jiedu Huayu Decoction on PI3K/Akt and AMPK Pathways

**FIGURE 8:**
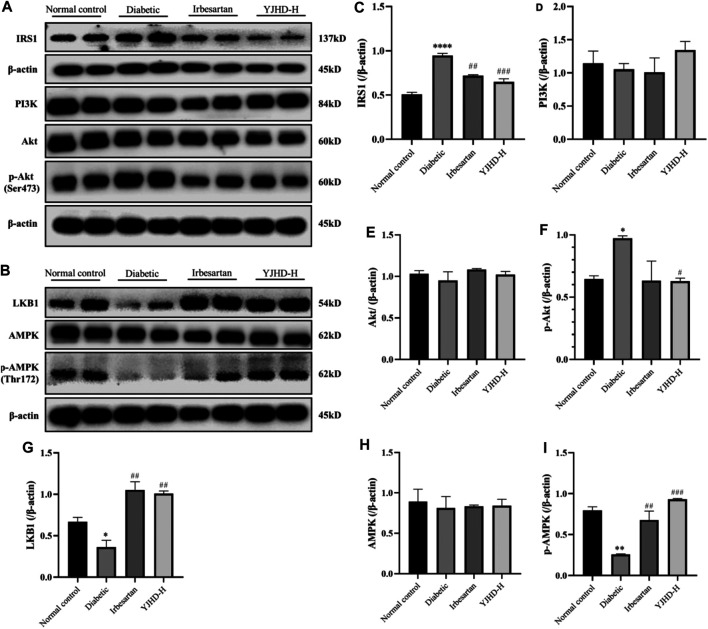
Effects of Yiqi Jiedu Huayu decoction (YJHD) on PI3K/Akt and AMPK pathways. **(A)** Protein levels of IRS1, PI3K, Akt, and p-Akt were detected by western blotting. **(B)** Protein levels of LKB1, AMPK, and p-AMPK were detected by western blotting. **(C–I)** Protein concentration analysis. Data are expressed as *p < 0.05, **p < 0.01, ***p < 0.001: compared with the normal control group, respectively; #*p* < 0.05 and ##*p* < 0.01: compared with the diabetic group respectively.

Considering that the mTOR pathway is mainly regulated by the PI3K/Akt and AMPK pathways, we examined the expression of proteins in the PI3K/Akt and AMPK pathways in the kidney. There was no significant change in PI3K and Akt in the diabetic group compared with the normal control group (*p* > 0.05) (Figures 8A,D,E), but IRS1 and p-Akt increased significantly (*p* < 0.05), indicating that the PI3K/Akt pathway was activated in the kidneys of DN rats, which was consistent with the activation of the mTOR pathway. Compared with the diabetic group, high-dose YJHD significantly reduced the expression of IRS1 and p-Akt and inhibited the PI3K/Akt pathway (*p* < 0.05) (Figures 8A,C,F), which was consistent with the inhibition of the mTOR pathway.

The AMPK pathway has a positive regulatory effect on autophagy, which is related to the inhibition of mTOR pathway activation. Compared with the normal control group, the expression of LKB1 and p-AMPK in the diabetic group was significantly decreased (*p* < 0.05), indicating that the AMPK pathway was inhibited in the kidneys of DN rats. Compared with the diabetic group, the expression of LKB1 and p-AMPK was significantly upregulated after high-dose YJHD treatment (*p* < 0.01) (Figures 8B,G–I), which promoted the activity of AMPK pathway.

## Discussion

As the major microvascular complication of diabetes mellitus, DN is one of the main causes of chronic kidney disease and ESRD (Molitch et al., 2004). Since the decreased renal function and pathological changes associated with ESRD are difficult to reverse, it is particularly important to develop effective early treatments. YJHD is composed of seven herbs. We identified fifteen different compounds by UPLC-QTOF-MS analysis. Studies have shown that ten compounds, including kaempferol and baicalein, can effectively improve diabetic nephropathy (Ahad et al., 2014; Sharma et al., 2020). Furthermore, seven compounds, including berberine and quercetin, have been shown to induce autophagy (Li et al., 2020; Qu et al., 2020). This is consistent with the overall effect of YJHD observed in this study. Therefore, these compounds may be potential components for the therapeutic effects of YJHD in diabetic nephropathy. In this study, we explored the renal protective effects of YJHD in DN and its underlying molecular mechanisms. We found that YJHD could ameliorate the symptoms associated with glucose metabolism disorders, such as polydipsia, polyphagia, polyuria, weight loss, and hyperglycemia, in DN rats. Increased urinary protein, serum creatinine, and urea nitrogen reflect glomerular filtration barrier impairment and reduced renal filtration function. In this study, we observed that YJHD reduced urinary protein, serum creatinine, and urea nitrogen in DN rats. This suggests that YJHD plays a role in improving renal function in DN.

The main pathological changes of DN include glomerular hypertrophy, basement membrane thickening, and renal fibrosis caused by accumulation of ECM in the mesangial area and tubulointerstitium (Palsamy and Subramanian, 2011; Han et al., 2017b). Accumulation of ECM in the mesangial region leads to diffuse and nodular mesangial expansion. With the development of DN, diffuse mesangial dilation gradually becomes a nodular accumulation in the mesangial matrix. These nodular deposits are known as Kimmelstiel-Wilson nodules, which are important pathological changes in late-stage DN (Tervaert et al., 2010). The gradual occurrence of renal fibrosis is an important factor for the continuous deterioration of renal function in diabetic patients and eventually into ESRD (Li and Zhang, 2017). In this study, we observed significant renal enlargement, mesangial expansion, basement membrane thickening, and renal fibrosis in the DN rats. In addition, collagen IV and fibronectin, the main components of the ECM, were significantly increased in the kidney. At the same time, we found that 12-weeks YJHD treatment alleviated the above pathological changes to varying degrees, reduced the expression of collagen IV and fibronectin in the kidney, and improved renal fibrosis. Kidney filtration function decreases with the progression of renal fibrosis. This leads to the accumulation of certain metabolic wastes, like Cr and BUN, which cannot be excreted by the kidneys and accumulate in the blood. Persistent high levels of Cr and BUN in the blood can damage other organs, including the brain. In this study, we observed that the serum Cr and BUN levels in the DN rats increased significantly, while YJHD decreased serum Cr and BUN levels. These results indicate that YJHD can alleviate renal pathological damage and improve renal filtration function in DN.

Glomerular visceral epithelial cells, also known as podocytes, form a glomerular filtration barrier together with the basement membrane and vascular endothelial cells. Unlike the latter two, podocytes not only participate in creating a mechanical barrier, but also build a charged barrier because the foot processes are negatively charged, which helps to retain small-molecule proteins in the blood (Lu et al., 2019). Fewer podocytes and increased functional impairment play an important role in the pathomechanism of DN (Lemley et al., 2002; White et al., 2002; Wiggins, 2007). Studies have shown that DN can cause podocyte hypertrophy, epithelial-mesenchymal transition, detachment from the basement membrane, and apoptosis, affecting podocyte morphology and quantity (Dai et al., 2017). Injured podocytes cannot maintain the integrity of the glomerular filtration barrier, which will further lead to continuous proteinuria and renal fibrosis and promote the progression of DN (Lu et al., 2019). To evaluate podocyte damage, we examined the expression of podocin and nephrin, proteins specifically expressed on the slit diaphragm of podocytes. Decreased expression of podocin and nephrin indicates podocyte damage and a reduction in normal podocytes. We found that the expression of podocin and nephrin in the kidney of the DN rats was significantly decreased, while YJHD significantly upregulated the expression of podocin and nephrin compared with the Diabetic group. This indicates that YJHD improves the damage to podocytes in DN.

**FIGURE 9:**
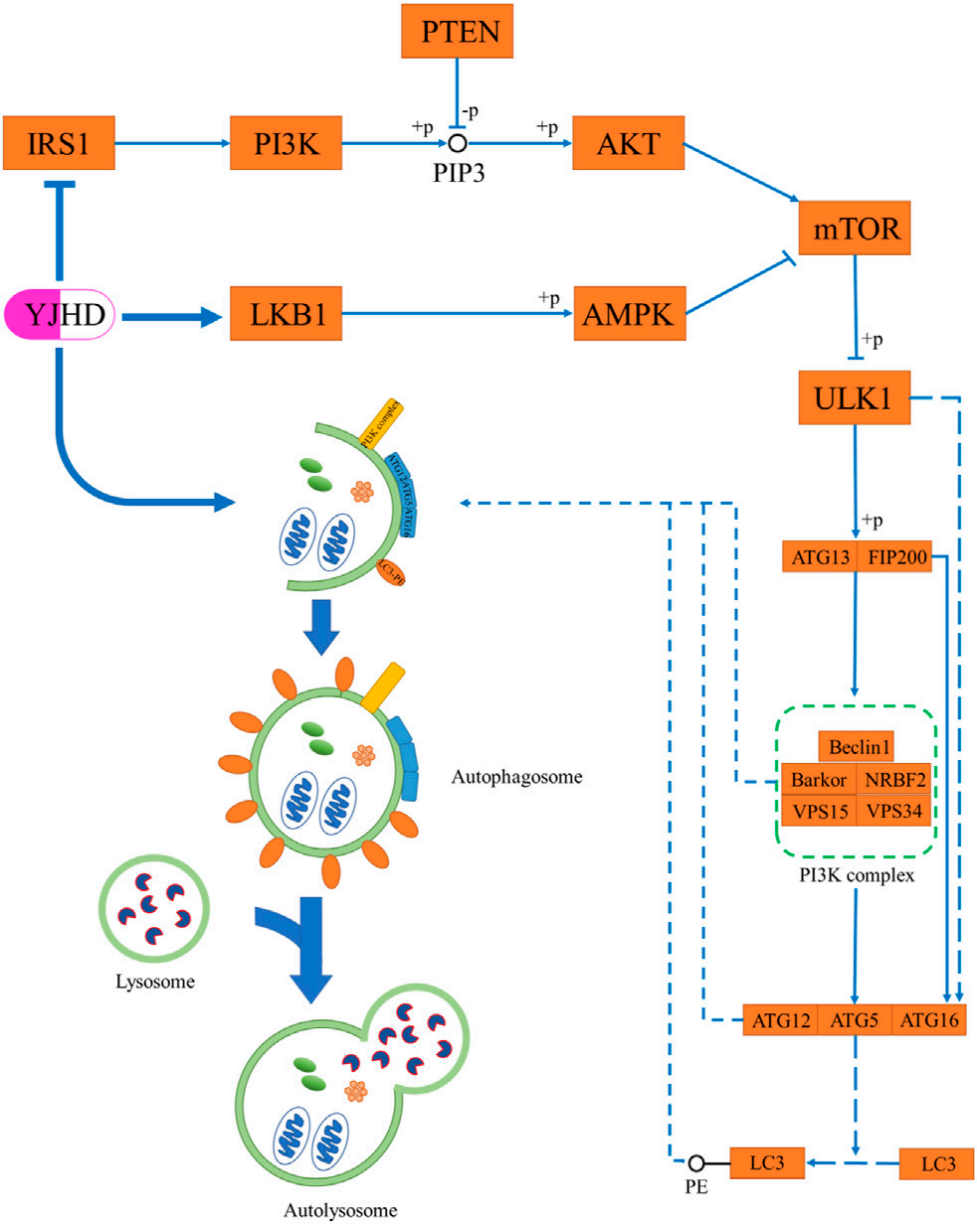
Schematic diagram of autophagy process.

As terminally differentiated cells, podocytes are difficult to regenerate when they are injured or apoptotic, and so the ability of podocytes to maintain their homeostasis in the face of stress stimuli is particularly important (Maezawa et al., 2015). Unlike other cells in the kidney, podocytes have a high basal level of autophagy, which is crucial to their homeostasis maintenance (Hartleben et al., 2010; Fang et al., 2013). *In vivo* and *in vitro* studies have shown in diabetic nephropathy, the level of podocyte autophagy is inhibited, and podocyte integrity is disrupted, thereby causing proteinuria and renal fibrosis. Promoting autophagy has been shown to attenuate podocyte injury and ameliorate renal fibrosis (Kawakami et al., 2015; Liu et al., 2016; Jin et al., 2019; Fan et al., 2020). Autophagy is a highly conserved intracellular lysosome-mediated protein degradation pathway that plays an important role in removing damaged or excessive organelles to maintain intracellular homeostasis and cell integrity (Yorimitsu and Klionsky, 2005). The formation of autophagosomes is at the core of the autophagic process, which includes four steps: trigger, nucleation, extension, and closure (Figure 9) (Ravanan et al., 2017). Phosphorylation of ATG13 and FIP200 by ULK1 is the key to triggering autophagy (Jung et al., 2009). Nucleation of autophagosomes depends on the PI3K complex, which is composed of Beclin-1, VPS15, VPS34, NRBF2, and Barkor (Liang et al., 1999; Kihara et al., 2001; Itakura et al., 2008; Sun et al., 2008). The formation of the ATG12-ATG5-ATG16 complex contributes to the coupling of LC3 with phosphatidylethanolamine (PE) to form LC3II and localize to autophagosomes, which is crucial to autophagosome extension. Therefore, LC3II is considered an important indicator for evaluating autophagosomes (Ichimura et al., 2000). After autophagosome formation, fusion with lysosomes leads to autophagy lysosomes. At this point, lysosomal enzymes begin to digest proteins or organelles encapsulated by autophagosomes (Yorimitsu and Klionsky, 2005). During this process, p62 is continuously degraded, which means that the level of p62 expression is negatively correlated with the level of autophagy (Bjørkøy et al., 2005; Pohl and Jentsch, 2009). Numerous studies have shown that the level of podocyte autophagy is inhibited in DN, and promoting autophagy is a promising potential mechanism for the treatment of DN (Kume et al., 2014; Tagawa et al., 2016). In this experiment, we observed that the levels of LC3II and Beclin-1 were significantly decreased in the kidneys of DN rats, while the p62 expression was increased, which indicated that autophagy was inhibited. Compared with the diabetic group, YJHD increased LC3B and Beclin-1 expression while decreasing p62 expression, indicating that YJHD promoted autophagic activity. It should be noted that the above results are the effects of YJHD on renal autophagy. Whether this result is accurate for podocytes will need to be further validated in subsequent cell experiments.

Studies have shown that overactivation of the mTOR pathway plays a key role in glomerular and tubular injury in DN. Selective inhibition of the mTOR pathway by rapamycin has renal protective effects (Noda and Ohsumi, 1998). The mTOR pathway is the main pathway to inhibit autophagy. The activated mTOR pathway phosphorylates and inactivates ULK1, making it unable to trigger autophagy (Hosokawa et al., 2009). In this study, we observed that the mTOR pathway was overactivated in the kidneys of DN rats, and p-ULK1 expression was significantly increased. Compared with the diabetic group, YJHD inhibited mTOR pathway activation and downregulated p-ULK1 expression. This suggests that YJHD can promote autophagy by inhibiting the mTOR pathway.

The activity of the mTOR pathway is mainly regulated by the PI3K/Akt and AMPK pathways. The activated PI3K/Akt pathway inhibits autophagy by activating the mTOR pathway (Inoki et al., 2002). Studies have shown that the PI3K/Akt pathway is overactivated in DN. Inhibition of the PI3K/Akt pathway can promote autophagy and reduce podocyte injury (Yang et al., 2020). This is consistent with the results observed in this study. IRS1 and p-Akt expression was significantly increased in the kidneys of DN rats, which indicated that the activity of the PI3K/Akt pathway was enhanced. The expression of IRS1 and p-Akt decreased after YJHD treatment, suggesting that YJHD can inhibit the activity of the PI3K/Akt pathway. AMPK is regulated by LKB1; in its low-energy state, AMPK is activated by LKB1. Activated AMPK inhibits the mTOR pathway by phosphorylating the TSC1-TSC2 complex (Gwinn et al., 2008). Therefore, the activated AMPK pathway has a role in promoting autophagy (Liang et al., 2007). Studies have shown that AMPK pathway activity is inhibited in DN, and the activation of the AMPK pathway by drugs can effectively alleviate kidney injury (Wang et al., 2018). In this study, we observed a significant decrease in LKB1 and p-AMPK expression in the kidneys of DN rats. Compared with the diabetic group, YJHD upregulated LKB1 and p-AMPK expression. This suggests that YJHD can activate the AMPK pathway. According to our results, YJHD can inhibit PI3K/Akt pathway activity and activate the AMPK pathway in the kidneys of DN rats. Therefore, we speculate that YJHD may inhibit mTOR pathway activity and ultimately promote autophagy by regulating the PI3K/Akt and AMPK pathways.

## Conclusion

In conclusion, our study shows that YJHD improves renal pathological damage and renal function in DN rats. This renal protective effect may be related to the downregulation of mTOR pathway activity through the PI3K/Akt and AMPK pathways, thus promoting autophagy and improving podocyte injury.

## Data Availability

The raw data supporting the conclusion of this article will be made available by the authors, without undue reservation.
